# New avenues for mechanochemistry in zeolite science

**DOI:** 10.1039/d1dt01440d

**Published:** 2021-06-17

**Authors:** Daniel N. Rainer, Russell E. Morris

**Affiliations:** School of Chemistry, EaStCHEM, University of St. Andrews North Haugh St. Andrews Fife KY16 9ST UK dnr2@st-andrews.ac.uk; Department of Physical and Macromolecular Chemistry, Faculty of Sciences, Charles University Hlavova 8 128 43 Prague 2 Czech Republic

## Abstract

Zeolites are a class of microporous materials with tremendous value for large scale industrial applications such as catalysis, ion exchange, or gas separation. In addition to naturally ocurring variants, zeolites are made synthetically using hydrothermal synthesis, requiring temperatures beyond 100 °C and long reaction times up to weeks. Furthermore, specific applications may require more sophisticated synthesis conditions, expensive reagents, or post-synthetic modifications. Some of these issues can be tackled by using the reemerged technique of mechanochemistry. In 2014, Majano *et al.* reviewed the space and outlined several possibilities for the usage of mechanical forces in zeolite chemistry. Since then the field has seen many more publications employing mechanochemical methodology to further and improve the synthesis and properties of zeolite materials. The usage ranges from the activation of raw materials, rendering the synthesis of the widely used catalysts much more economical in terms of duration, atom efficiency, and production of waste, to post-synthetic modification of the materials leading to improved properties for target aplications. We present a short review of the advances that have been reported recently, highlight promising work and important studies, and give a perspective of potential future endeavours.

## Abstract 2

Recent advances of mechanochemical methods in the field of zeolite science are reviewed and their future potential highlighted.

## Graphical abstract

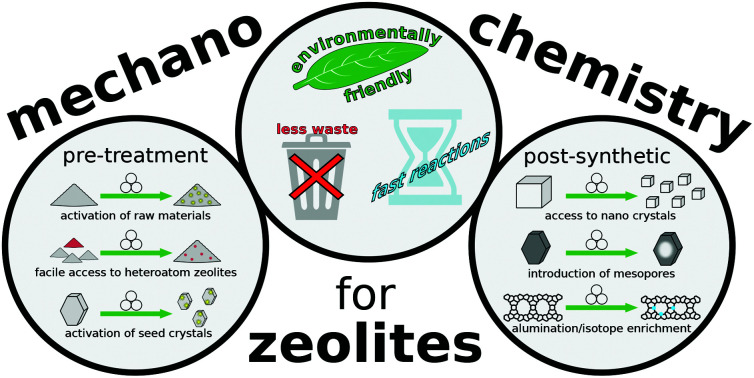

## Introduction

Over the last few decades, mechanochemistry has seen an increase in popularity, especially as a possible solution for various drawbacks of conventional syntheses and processes in several branches of chemistry. Commonly, chemical reactions and transformations are carried out in solution, producing not only the desired product but also considerable amounts of waste solvent. This is particularly concerning in organic chemistry where often toxic and carcinogenic halogenated solvents are used. Even compounds that are generally considered benign like ethanol and wastewater, which are more prevalent in inorganic chemistry and materials science, may have to be disposed of as special waste. A promising solution for this widespread issue is employing mechanochemical synthesis methods that only require minute amounts of solvent and can sometimes be conducted with no liquid at all. Additionally, applied mechanical forces can open up new reaction pathways that are inaccessible using conventional synthesis methods. Several excellent reviews of mechanochemical synthesis are available, covering many facets from fundamental and general concepts^1–11^ to specific applications in various fields such as main group chemistry,^12^ organic chemistry,^13^ catalysis,^14^ and materials chemistry,^15–20^ in particular porous materials^21,22^ such as metal–organic frameworks (MOFs),^23–26^ zeolitic imidazolate frameworks (ZIFs),^27^ and porous organic materials such as covalent organic frameworks (COFs).^28^ The potential of mechanochemical methods for modern zeolite chemistry has been discussed previously by Majano, Pérez-Ramírez, and co-workers in their review in 2014.^29^

Zeolites are one of the most important classes of porous materials. They find use in several large-scale industrial processes, for example as catalysts,^30,31^ and membrane materials for gas separations.^32^ Structurally, these microporous aluminosilicates are comprised of tetrahedral TO_4_ units, where the T atoms (Si, Al) are connected *via* oxygen bridges. The wide range of possible T–O–T angles gives rise to a plethora of resulting frameworks, which differ most importantly in their pore structure and connectivity. Zeolites occur naturally but can also be made in the laboratory by synthesising these materials under hydrothermal conditions.^33,34^ Over time, researchers have advanced and optimised synthesis conditions of this hydrothermal process and together with an expansion of the available pool of T atoms (Ge, P, Sn, Ti, *etc.*) there are more than 250 unique framework types recognised today.^35^ Currently, there are only a handful of cases in which a synthetic zeolite was obtained through means other than the described hydrothermal method. For the most important class of zeolite materials, the aluminosilicates, only a specific combination of high temperature, pressure, long reaction time (often days up to months), appropriate aqueous medium (typically highly alkaline), the presence of a suitable structure directing agent and adequate source materials in a precise ratio leads to pure products. These conditions have to be met to form a zeolite phase, which is thermodynamically less favoured than denser polymorphs such as quartz or cristobalite.

**Fig. 1:**
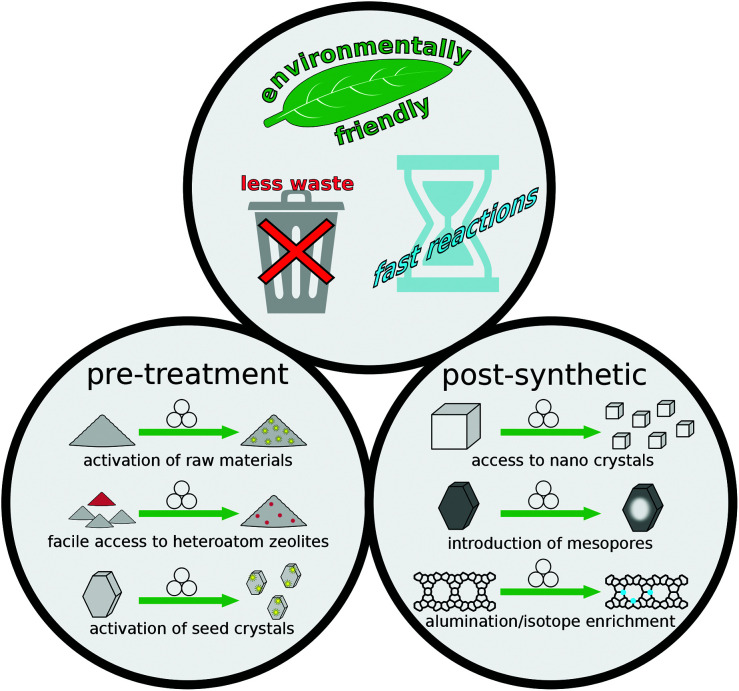
Schematic overview of several advantageous avenues for mechanochemical methods in zeolite science.

Spurred on by the success of mechanochemical methods in the related fields of MOFs, ZIFs, and COFs, the number of studies published from zeolite researchers has continuously increased. These works include advances of previously reported concepts such as mechanical activation of reagents, as well as systematic investigation of influential parameters during mechanical treatments and many more topics (Fig. 1 and Table 1).

**Table 1:** Overview of mechanochemical methods in zeolite science and their advantages

	Application of mechanochemistry	Advantages
Pre-treatment	Activation of raw materials	Fast synthesis, solvent-free
	Heteroatom zeolites	Fast synthesis, access to cheap precursors
	Milling of seed crystals	Fast synthesis, well-controlled synthesis conditions
Post-treatment	Heteroatom zeolites	Avoiding wet chemistry (impregnation)
	Milling + recrystallisation	Well-controlled (nano) crystal size
	Modification of textural properties	Particle size reduction, formation of meso-/macropores

A particular issue for zeolite science that has not been satisfactorily explained is why there are so few examples of pure mechanochemical synthesis where the final material is prepared from starting materials just using mechanical energy. Often the preparation of zeolites involves a mechanochemical pre-treatment and a further heating step before the required material is formed. Nevertheless, this can be beneficial compared to hydrothermal synthesis.

The following perspective shall provide a summary of recent contributions and the current state of the area. Earlier reports, which have already been described in the review by Majano *et al.*,^29^ are only included where additional context was deemed necessary. We try to highlight important advances and the potential of published works but are also pointing out some of the challenges that mechanochemistry and its methods are faced with when tackling issues in zeolite science, with the overall goal of explaining where mechanochemistry can make major contributions to the field, and where work needs to be done to maximise its impact.

### Mechanochemistry prior to crystallisation

**Fig. 2:**
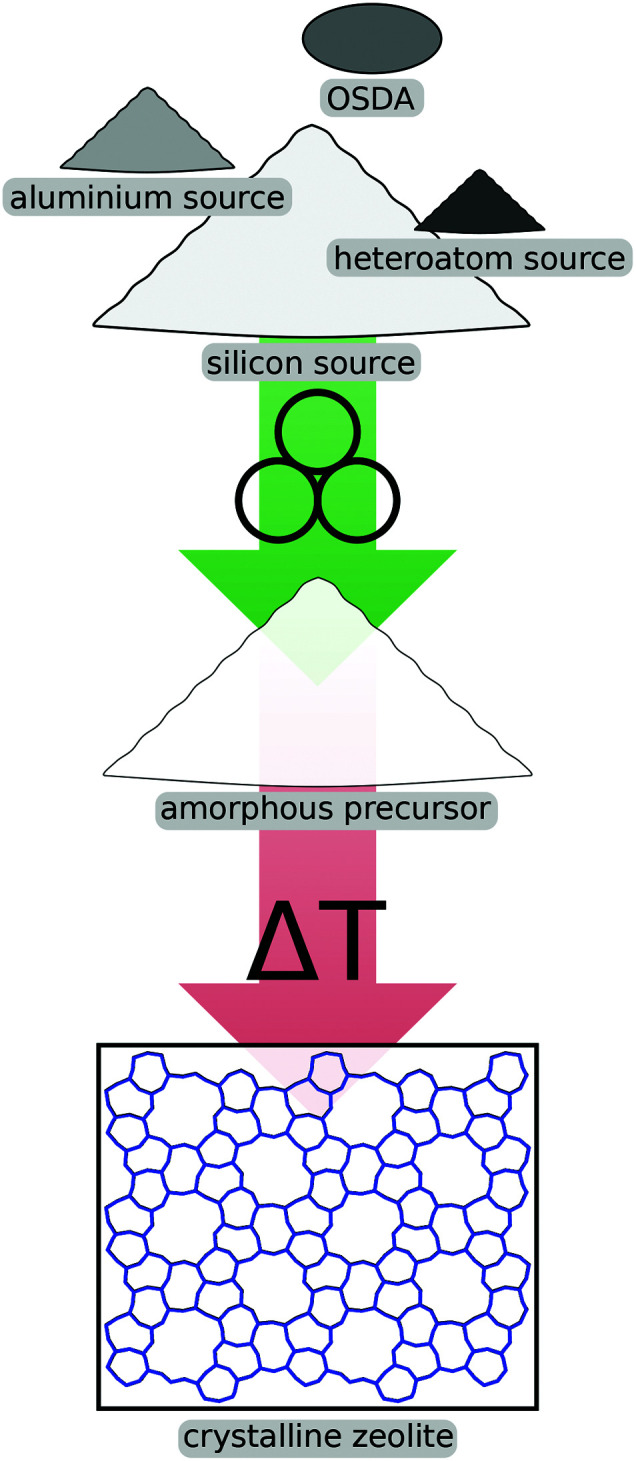
Schematic illustration of mechanochemical pre-treatment of reagents prior to crystallisation.

One of the most promising avenues for mechanochemical methods in the field of zeolites is the activation of reagents prior to crystallisation, schematically shown in Fig. 2. Using such a pre-synthesis step, industrially relevant zeolites like ZSM-5 (which is given the international zeolite association topology code **MFI**) or zeolite Y (**FAU**) can be prepared much more economically due to improved kinetics in the ensuing crystallisation. Generally, one can distinguish between methods where the mechanical forces are exerted on the raw precursor compounds like fumed silica or aluminium hydroxide from approaches which involve seed crystals of the desired zeolite phase. This methodology has also been successfully extended to include heteroatom sources which lead to materials with exciting properties for specific applications.

### Mechanical activation of raw materials

Mechanochemical treatment of zeolite synthesis reagents has been used by several groups to shorten synthesis time and reduce the amount of required solvent and organic structure directing agent (OSDA). Starting in the 2000s, Gordina, Prokof'ev, and co-workers published a series of studies on the effect of mechanochemistry on the synthesis of zeolite A (**LTA**).^36–40^ Remarkably, their earliest report^36^ is to date the only published result of mechanosynthesis of a zeolite without the explicit need for subsequent crystallisation at elevated temperature for the zeolite phase to form. A following calcination step did, however, increase crystallinity of the product and residual starting material was further transformed into the zeolite. In consecutive works, they discussed the viability of inexpensive starting materials like metakaolin and the parameters influencing phase selectivity.^41^ Impurity phases were found to be favoured when using more concentrated sodium hydroxide solutions as crystallisation media (sodalite), calcination temperatures beyond 600 °C (nepheline) or orthorhombic sodium aluminate, instead of its cubic or tetragonal forms (sodalite).

The last decade also saw the advent of solvent-free synthesis of zeolites as introduced by Xiao and his group. Their work has been summarised recently, where all synthesised materials as well as the understanding of the process so far are described comprehensively.^42–44^ In this method, a grinding or milling step of the solid raw materials precedes the crystallisation in conventional autoclaves, but in absence of a solvent, with temperatures typically ranging from 150 to 200 °C. It was established that a certain, albeit small amount of water is still required for successful synthesis. This could, however, be introduced *via* the precursors themselves such as hydrated sodium silicate, *e.g.*, Na_2_SiO_3_·9H_2_O. Similarly, the mechanochemical activation requires a certain amount of energy, *i.e.*, a certain minimum milling time needs to be exceeded. The versatility of this approach is astounding, as zeolites with various frameworks have been prepared since the original publication in 2012.^45^ The procedure has also been extended to include aluminophosphate,^46,47^ silicoalumino-phosphates,^46,48,49^ and heteroatom containing zeolites.^50–53^

Several groups have recently tried to elucidate the underlying mechanism of these new zeolite syntheses. In the study on zeolite A (**LTA**), Xiao and co-workers were able to show that after manual grinding of the solid reagents a zeolite phase formed after heat treatment for 3 h.^54^ The crystallinity can be increased by longer treatment for up to 5 h, which is still faster than most conventional synthesis methods. SEM analysis revealed the crystallisation starting on the surfaces of amorphous particles obtained after the mechanochemical pre-treatment. Using several techniques such as UV-Raman spectroscopy, they also observed the initial formation of a small number of four-ring species, where four T atoms (T = Si, Al) are connected by oxygen atoms into a ring, during milling which continued during the early stages of heating. The other rings that make up the framework of LTA, consisting of six and eight T atoms, are only observed at longer times at elevated temperature.

**Fig. 3:**
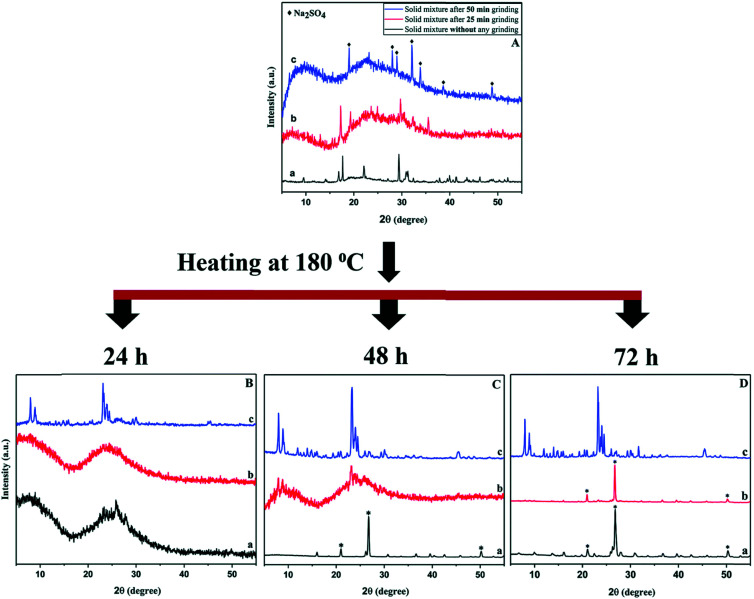
PXRD patterns of samples prepared without milling (a, bottom, black), 25 min (b, middle, red) and 50 min ball milling (c, top, blue). The top panel shows materials after the mechanochemical treatment, the bottom panel the respective products after heating in an autoclave for various time periods, showcasing the importance of sufficient mechanical activation of the precursors prior to crystallisation at elevated temperature in the synthesis of ZSM-5. Reproduced from ref. 57 with permission from the Royal Society of Chemistry.

Nada *et al.* investigated the mechanistic effects of the mechanochemical treatment in the solvent-free synthesis using the **MFI** system as a case study.^55–57^ For manual grinding with mortar and pestle, a minimal contact time of 15 min was found to be necessary for a thermally stable crystalline end-product with **MFI** framework. Using a ball mill, this can be shortened to 5 min with the added benefit of greater reproducibility. In either case, mechanical force of sufficient energy is required to be able to release water of the hydrated raw materials to form reactive intermediates. In addition, an ammonia source in the form of a halide salt (*e.g.*, NH_4_Cl) appears to be equally crucial. The products of the mechanochemical reaction are the sodium halide salt, ammonia, which provides the basic environment for the ensuing crystallisation in the autoclave, and an (alumina-)silica species. The compounds used as silicon and aluminium source were sodium silicate nonahydrate, and aluminium hydroxide or aluminium sulfate, respectively. In experiments including the sulfate salt, manual grinding was found to be insufficient and only the higher energy in a ball mill resulted in desired product, although only after a milling time of 50 min, once again illustrating the required minimal duration of the procedure (see Fig. 3). Noteworthy is that these, latter syntheses were conducted without the use of a structure-directing agent, and two zeolite phases with frameworks **MFI** and **MOR** were obtained. Pure phases of either zeolite could be achieved by adjusting the Na/Al ratio of the solid mixture.

Zeolites with the **MOR** framework were also the focus point of Kornas *et al.* who similarly investigated the influence of several parameters for pre-synthetic ball milling of raw materials.^44,58^ Their results show once more, that manual grinding with mortar and pestle is insufficient as a pre-treatment. When utilising the higher energy of a ball mill, however, phase pure mordenite may be obtained. The authors noted a difference when comparing neat grinding (no added liquid to the milling jars) to wet or liquid assisted grinding (LAG). Using water resulted in some extra-framework aluminium species, which were not observed under dry conditions. Important to note is that for the neat grinding experiments, some small amount of water had to be added during the subsequent heat treatment in order to obtain the zeolite product, in agreement with previous reports.

Combinations of mechanochemical activation of starting materials with alternative techniques for crystallisation have also been reported. The necessary amount of water for transformation of ball milled precursors into a zeolite can for example be supplied as steam, showcased with amorphous reagents and in the absence of an OSDA^59^ as well as in an inter-zeolite transformation of **FAU** into **CHA**.^60^ Microwave radiation has been used as energy source for crystallisation of AlPO_4_-5 (**AFI**), where direct comparison of manual grinding with ball milling showed higher phase purity using the latter method.^61^

### Access to heteroatom zeolites *via* mechanochemistry

Altering zeolite materials to better accommodate the needs for specific applications has led researchers to include heteroatoms (T-atoms other than silicon or aluminium) into the framework. Typically, this is accompanied with a change in synthesis conditions since the presence of a new species in the used gel can induce an additional structure directing effect. Sometimes this is an intended effect in order to obtain zeolites with a new framework, with the typical example of germanosilicate which have opened the door for a whole range of new frameworks.^62,63^ In other cases, however, the presence of heteroatoms can lead to dense or amorphous phases, a mix of several products, or the target framework but without the incorporation of the heteroelement. For titanosilicates, the use of alkoxides, mainly Ti(OBu)_4_, as reagent usually gives good results, with the main drawback of high cost, compared to readily available inorganic precursors like TiO_2_.

In the last two decades, several studies have shown that pre-synthetic mechanochemically treated metal oxide reagents can be used instead of expensive organic analogues. Yamamoto, Borjas Garcia, and Muramatsu reported the successful synthesis of titanosilicate TS-1 (**MFI**)^64–66^ and Ti-beta (*BEA)^67^ from bulk titania and silica, which were ball milled for 36 h and used as a combined T-atom source in a following conventional hydrothermal synthesis of the respective zeolites using tetrapropylammonium hydroxide (TPAOH) as OSDA. Using this concept, significant reduction of associated cost was achieved by using the cheaper bromide salt of TPA.^68,69^ Recently, the successful synthesis of a titanosilicate zeolite with **MWW** framework combined the described milling pre-treatment with a dry-gel conversion method to produce highly efficient catalysts for the epoxidation of 1-hexene.^70^

**Fig. 4:**
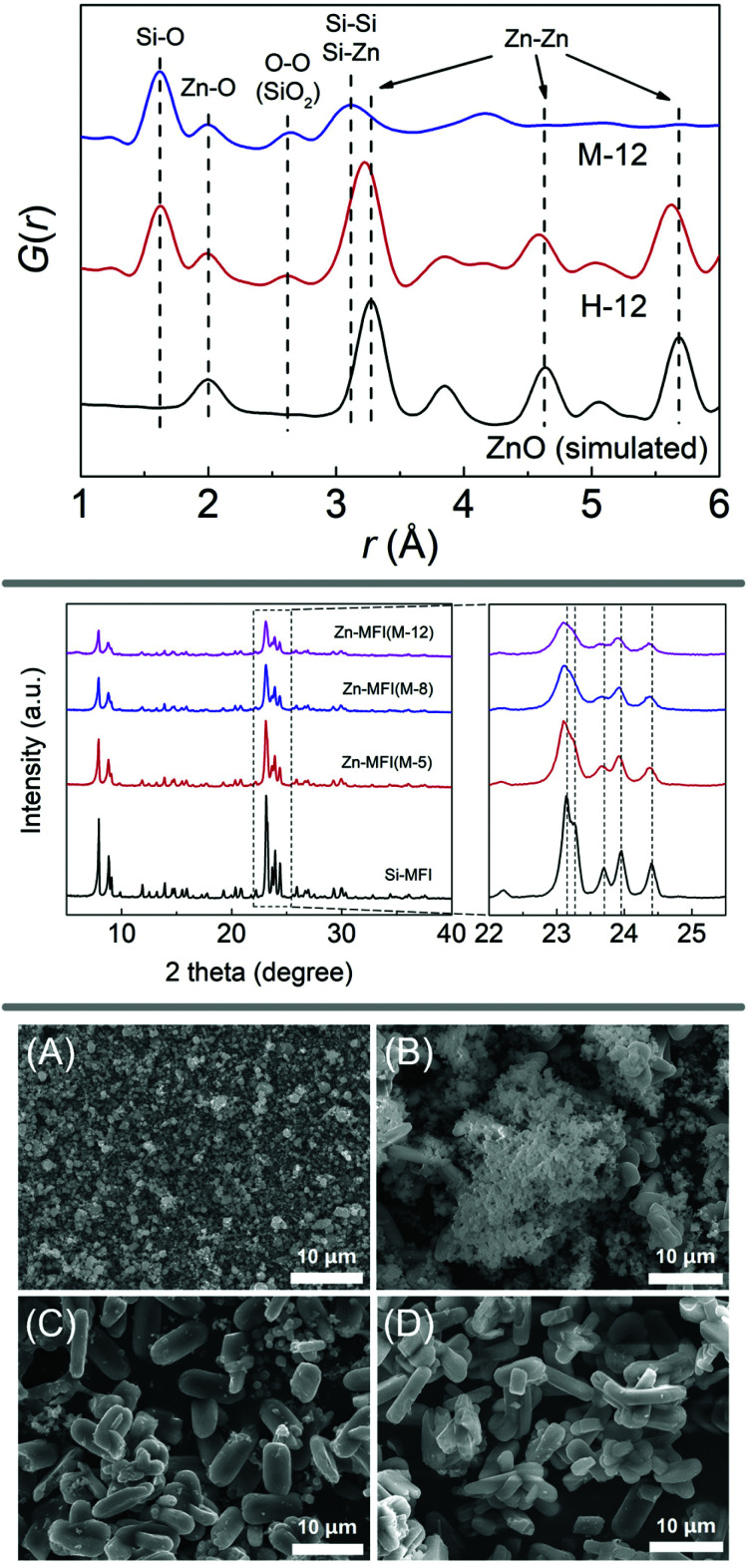
X-ray diffraction and scanning electron microscopy analysis of a zincosilicate zeolite with MFI framework. The top panel shows PDF analysis of the ball milled precursors, evidencing a mixed silicon-zinc oxide. PXRD patterns are shown in the middle, comparing experiments with varying amounts of zinc (middle panel). The bottom panel shows SEM images of ball milled precursor (A) as well as samples obtained after 1, 2, and 3 days of hydrothermal synthesis (B, C, and D, respectively). Reproduced from ref. 76 with permission from Elsevier, copyright 2019.

In a similar manner to the fabrication of the Ti/Si-oxide composite precursor, researchers have prepared Sn/Si-, Mn/Si-, Ga/Si-, and Zn/Si-oxide precursors for the synthesis of zeolites Sn-beta (*BEA),^71^ Sn-silicalite-1 (**MFI**),^72^ Mn-MCM-41,^73^ Mn-silicalite-1,^74^ Ga-silicalite-1^75^ and Zn-silicalite-1,^76^ respectively. PDF analysis, SEM images and PXRD patterns of the study investigating zinc-containing **MFI**-type zeolite are shown in Fig. 4. Further extending the methodology to mixing silica with oxides of alkaline earth metals, Yamamoto *et al.* were successful in obtaining new materials with interesting structures, such as a novel calcosilicate,^77^ or layered^78^ and microporous^79^ strontosilicates.

In the most recent example, aluminium and iron were incorporated into **MFI** zeolite, resulting in durable catalysts for the DTO (dimethyl ether-to-olefin) reactions.^80^ The authors determined once again the optimal conditions for all necessary steps (milling, ageing, hydrothermal synthesis) and succeeded in the preparation of catalytically active materials without often occurring impurity phases like extra-framework species of iron.

The results of these reports demonstrate the great potential of mechanochemical methods for reliable, fast, and cost-efficient introduction of heteroatoms into zeolites, which show comparable or even increased performance in catalysis or ion-exchange applications. The importance here lies in the ease of preparation of the materials as well as a potentially significant cost reduction, due to cheap(er) raw materials that can be used as well as the avoidance of by-products, thus increasing the efficiency of the synthesis itself.

### Mechanical activation of seed crystals

Studies on the mechanochemical treatment of seed crystals are mainly focused on their applicability for an accelerated crystallisation time. In one of the earliest published works, Valtechev *et al.* demonstrated this concept on zeolite Y (**FAU**),^81^ by first synthesising the zeolite *via* hydrothermal route, then exposing the material to mechanical forces in a planetary ball mill and finally using the product as seed crystals for subsequent syntheses. The treatment was shown to be most effective when the seeds and gel had been milled together, due to the high dispersion of seed particles throughout the gel medium. Direct comparison of employing raw seed crystals and mechanochemically treated seeds validated the authors expectations, effectively reducing the synthesis time from 30 h to only 4 h. The authors also observed that milling for longer than the optimised time of 15 min, resulted in the appearance of a second zeolite P (**GIS**) phase, which eventually becomes the only product after milling for more than 45 min.

The approach of utilising milled seed crystals has also been used by Xu *et al.*, who were able to drastically shorten the synthesis time for zeolite **DDR** membranes.^82^ Typically, this small-pore zeolite would need several weeks for successful crystallisation. When using milled seeds, however, this may be shortened to merely several hours. Not only did this improved synthesis succeed in conventional oven-based protocols but was also applicable for the growth of **DDR** membranes. The so prepared membranes were used for the separation of a CH_4_/CO_2_ mixture and showed improved performance compared to other **DDR**-based membranes, however, are still not competitive with other small pore zeolite-based materials. Nonetheless, the presented advantages of using ball milled seed crystals with respect to synthesis time as well as the resulting potential for growing membranes of desirable low thickness demonstrate once more the importance of mechanochemistry.

**Fig. 5:**
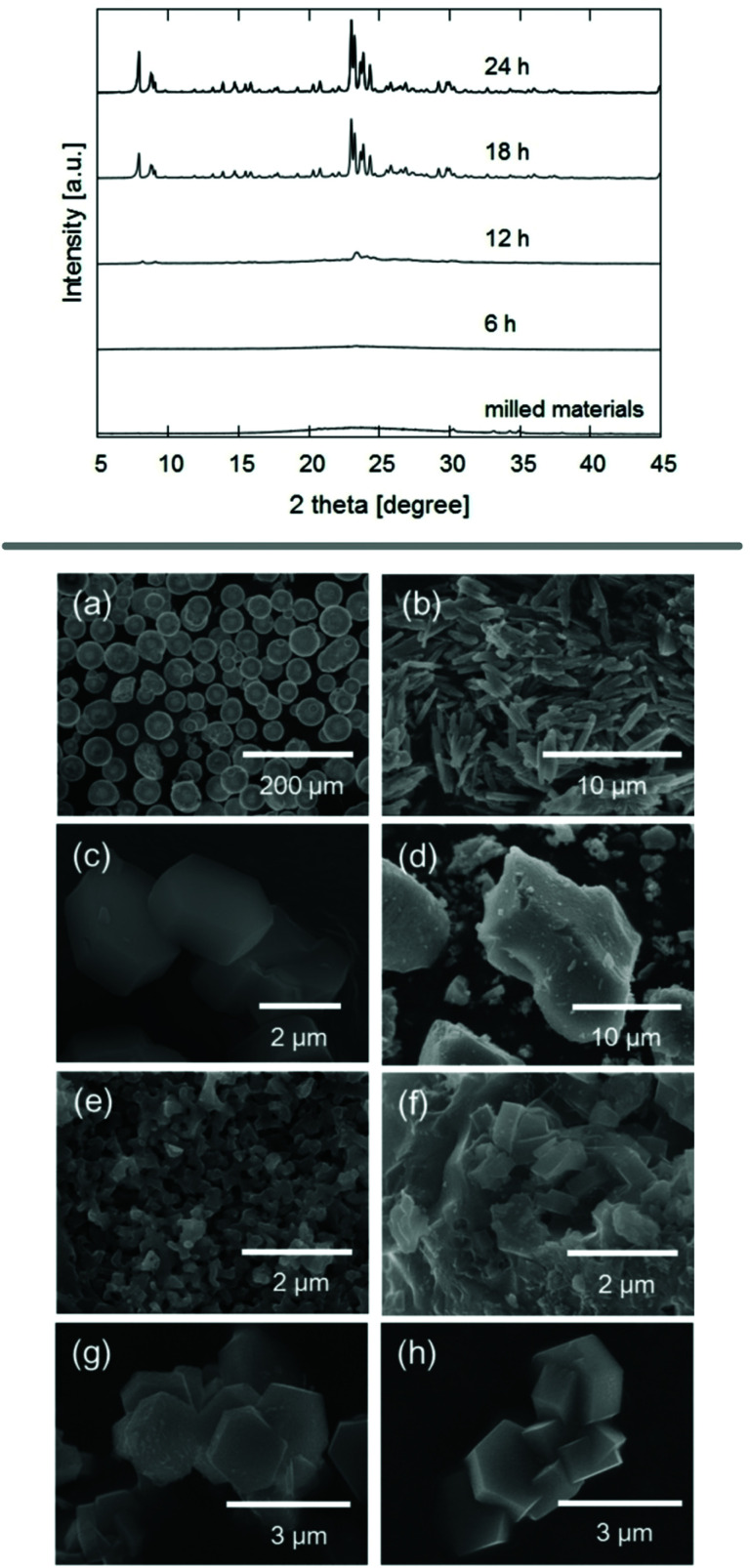
PXRD patterns and SEM images of experiments using ball milling for the treatment of reagents and zeolite seed crystals prior to steam treatment. SEM images show the precursors silica powder (a), sodium aluminate (b) and ZSM-5 seed crystals (c), as well as materials obtained after milling (d) and final products after 6 h (e), 12 h (f), and 18 h (g) of steam treatment. Reproduced from ref. 83 with permission from Wiley-VCH, copyright 2017.

Seed crystals may not only favour the formation of a specific framework, but also eliminate the requirement for an additional OSDA. Such an approach has been demonstrated on zeolite ZSM-5 by using mechanochemically pre-treated seeds to direct the formation of the desired **MFI** framework.^83^ After milling silicon and aluminium reagents and seeds for 1 d, a steam treatment resulted in pure phase ZSM-5 after only 24 h, demonstrated with PXRD and SEM shown in Fig. 5. The possibility of synthesising zeolites without the need for solvent or expensive organic additives is a very promising new tool for synthetic zeolite chemists.

Exposing seed crystals to mechanical forces prior to their use in a synthesis can also help to suppress the formation of impurity phases. During the preparation of zeolite membranes with **CHA** framework, Jiang *et al.* tried to avoid a secondary **MER** phase, which spersisted even when using seed crystals.^84^ However, a wet ball milling treatment of those seeds for 3 h resulted in a pure phasic product. This was ascribed to the reduced particle size and lattice defects, yielding highly crystalline **CHA** particles, which also showed improved performance in the dehydration of organic solvents under acidic conditions.

Besides the above reasons for the success of milled seeds in the synthesis of zeolites such as increased number of small particles, hence more nucleation sites, Zhang *et al.* have shown that mechanochemical treatment of seed crystals in a mill forms oxygen radicals that are beneficial for the crystallisation.^85,86^ The effect of radicals on zeolite synthesis had been reported earlier by the same group,^87^ where UV radiation or a reagent were necessary to generate the radical species. In their more recent work, the authors show the much more economical possibility of radical formation through mechanochemical means, by ball milling of **LTA**, silicalite-1, and beta seed crystals. The authors observe a similar trend to other reports, where optimal conditions need to be established to find a compromise between particle size decrease and generation of radicals on their surface on the one hand and amorphization of said particles on the other.

The identification of these radicals on the surface of ball milled seed crystals is a very important result in the quest to gain a complete understanding of the underlying mechanisms in mechanochemically assisted syntheses. So far, no studies have been conducted to investigate potential radical formation in mechanochemical activation of raw materials (compare above section on “Mechanical activation of raw materials”), but the availability of radicals might be a key parameter for the success of such experiments.

### Post-synthetic mechanochemical treatment of zeolites

Zeolite crystal size plays a crucial role for applications in catalysis and separation, where small, nanosized particles can outperform materials with dimensions on the micrometre scale.^88^ Although synthesis recipes exist which directly produce nanocrystalline materials, post-synthetic modification is usually necessary to obtain the desired dimensions and especially a uniform size distribution.^89^ Mechanochemical treatment of the hydrogel used for the hydrothermal synthesis of zeolites has been shown to influence the crystal size of the resulting material, amongst other properties.^90^

### Post-synthetic incorporation of heteroatoms

Including heteroatoms in a zeolite is an important tool for their functionalisation and can be achieved during synthesis itself as outlined above. Another possibility is a post-synthetic treatment of the material, for example, by impregnation techniques. Using mechanochemistry, Hammond *et al.* have successfully incorporated tin into beta zeolite, creating a catalytically active material which can be used in oxidation reactions.^91,92^ Conventionally prepared zeolite beta was first dealuminated under acidic conditions in order to create empty T sites in the framework. These are filled by incorporation of Sn in a ball mill, using tin acetate as a reagent. A subsequent calcination removed residual organic species and results in a fully connected structure. Recently, Schüth and co-workers investigated the mechanism of this mechanochemical reaction in more detail.^93^ The authors were able to confirm the previously stipulated hypothesis about “silanol nests” formed during the dealumination step and the subsequent regeneration of the framework by incorporation of tin ions. Employing tin diacetate as the tin source, the divalent Sn^2+^ cation is oxidised to the tetravalent and tetrahedral Sn^4+^ species as expected for a framework T atom. Using this specific reagent has the additional advantage of being small enough to reach available empty sites within the particles, which is inhibited when using the larger tetraacetate salt.

### Mechanochemically assisted recrystallisation

**Fig. 6:**
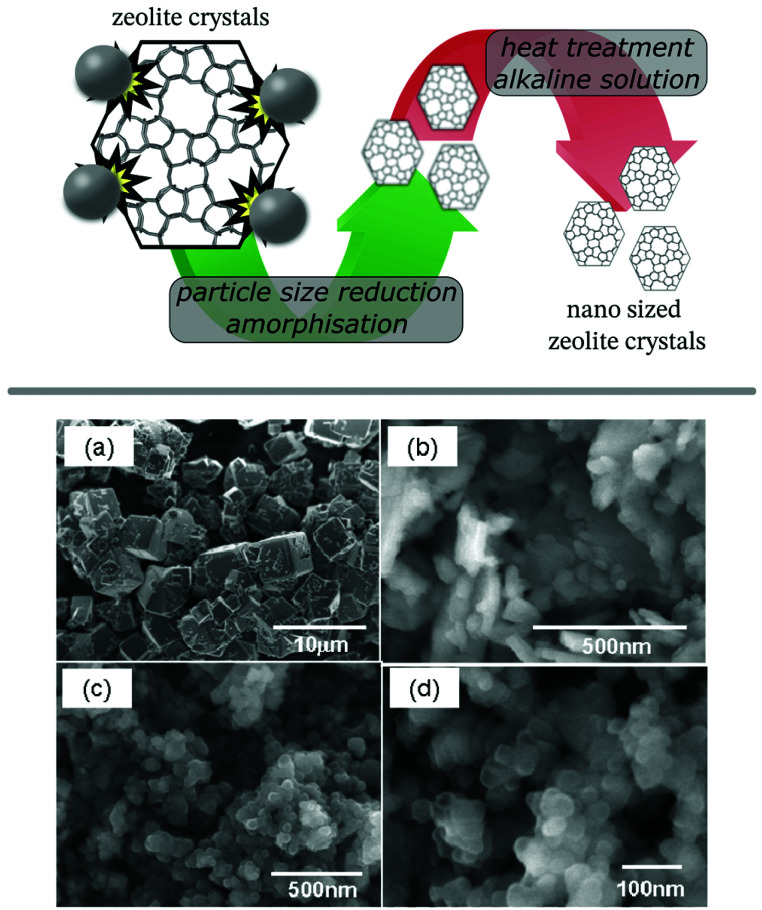
Schematic illustration of the preparation of nano sized ZSM-5 zeolite crystals by ball milling and subsequent recrystallisation (top panel) and SEM images of zeolite A (**LTA**) crystals: pristine (a), post milling (b) and after subsequent recrystallisation (c and d). Adapted and reproduced with permission from ref. 97 Copyright (2011) American Chemical Society.

Post-synthetic particle size reduction is typically performed by grinding or milling of a material, however, when performed on zeolites this has shown to result in considerable amorphization.^94,95^ A consecutive recrystallisation stage would be able to recover the crystallinity of the sample, but this simple method can result in unwanted particle growth, defeating the purpose of the mechanochemical treatment. Addressing this problem, the group around Okubo and Wakihara used dilute (aluminium-)silicate solutions as recrystallisation medium for milled zeolite crystals (ZSM-5, zeolites A, X, and beta) to ensure low crystal dimensions and high temperature stability (Fig. 6).^96–100^ This methodology was also employed for SAPO-34, recrystallised from either a freshly prepared aluminosilicate solution or the mother liquor of the original synthesis.^101^ A more concise procedure was presented as an *in situ* milling and recrystallisation method, using highly alkaline solutions during the milling procedure. The method was showcased on zeolite A (**LTA**), which was obtained as fully crystalline nanoparticles with an average size of only 66 nm.^102^ This one-step process, however, requires a modified ball mill setup to endure the high pH values of the used recrystallisation medium. In an alternative approach, the same group utilised their tubular reactor, to speed up the recrystallisation process. The reactor was originally developed for ultrafast as well as continuous synthesis of zeolites in mere minutes due to its rapid heating capability.^103^ Despite going back to a two-stage process, the advantage of combining a milling procedure with the tubular reactor results in a fast and continuous setup. The authors demonstrated the feasibility of this concept on SSZ-13 (**CHA**), AlPO_4_-5 (**AFI**),^104^ mordenite (**MOR**),^105^ and **CON**-type zeolite^106^ yielding nanosized and highly crystalline products after only 10 min of contact time in the reactor. The methodology was also extended to inter-zeolite conversion (IZC) in the synthesis of **AFX** zeolite from seed crystals with **FAU** framework.^107^ Establishing optimised conditions for each stage is crucial for the success of the procedure, recently illustrated on the SSZ-13 example.^108^ Using a low temperature (100 °C) recrystallisation initially, followed by the short high temperature treatment, enables optimal control over particle size and improved the thermal stability of the crystals, a vital parameter for catalytic applications like the selective catalytic reduction of NO_*x*_ (ammonia-SCR).

The recrystallisation technique has also found application in the selective formation of **MOR** phase from a naturally occurring zeolite sample containing three different phases (**MOR**, **HEU**, and quartz).^109^ Exerting mechanical forces on the raw sample and subsequent recrystallisation from an alkaline sodium silicate solution resulted not only in higher crystallinity of the entire sample but also produced **MOR** as the major phase. This result hints at great potential for enhancement of phase purity by selective recrystallisation promoted *via* mechanochemistry.

### Modification of textural properties using mechanochemistry

Among the early studies of how mechanochemical treatment affects zeolite properties like crystallinity, surface area and porosity are the contributions by Kosanović *et al.*^110–112^ and Zielinski *et al.*^95^ Building on this knowledge, a recent contribution focused on the industrially relevant zeolite H–Y, investigating the effects of different conditions during ball milling on material properties.^113^ Likewise, several studies on the effects of milling on the naturally occurring zeolite clinoptilolite were recently conducted.^114–116^ All of these works are in good agreement, highlighting the importance for careful selection of correct conditions depending on the desired outcome.

A study on the influence of altered surface properties of commercial zeolite 4A for the capture of CO_2_ serves as an example for potentially insufficient optimisation.^117^ In this case, only one set of parameters (350 rpm, 12 h) was used in the mechanochemical treatment using a planetary ball mill. Under these conditions, not only was the particle size smaller but the surface area of the resulting material also decreased (from ca. 27 m^2^ g^−1^ to 18 m^2^ g^−1^), and its CO_2_ uptake was reduced. However, as mentioned above, literature suggests that decreasing duration or frequency of the milling operation could help retain or even increase the available surface area. Such work would benefit from testing an expanded set of milling conditions, as otherwise a false sense of the inapplicability of mechanochemistry may be conveyed.

The decrease of particle size by ball milling has recently been utilised in the fabrication of a dye rejecting membrane, coated with nano sized zeolite Y.^118^ Although no direct comparison of membranes coated with zeolite both prior and after the milling operation were conducted, both zeolite samples were characterised and their properties assessed. The observed increase in zeta potential as well as the higher surface area are known to be beneficial for the target application and the final products performed well in the separation of dye molecules for an aqueous medium.

Equally important to the conditions of a mechanochemical treatment is at which point within the overall process the mechanochemical step is incorporated. An example of this has been reported on zeolite Cs-X (**FAU**), comparing milling before and after the ion-exchange procedure from sodium to the caesium form.^119^ The former leads to a material with comparable N_2_ adsorption behaviour than the parent zeolite Na-X. The observed lower PXRD intensity was attributed to the higher X-ray absorption coefficient of caesium. In contrast, the product of first ion-exchange and ball milling thereafter exhibits significant reduction in surface area, micropore volume and crystallinity. At first sight these results may seem to indicate an inferior catalyst for the side-chain alkylation of toluene, however, it outperformed the material that was milled prior to the ion-exchange. This could be explained by an increase of the number of active basic sites, resulting from a mechanochemical reaction of the zeolite itself with already present CsO species.

Post-synthetic mechanical treatment of the zeolite can also be used to improve catalytic performance through selective amorphization of the external surface, thus deactivating acid sites which do not benefit from the additional shape-selectivity of the pore system. This has been demonstrated by Inagaki *et al.* by ball milling zeolite ZSM-5, which resulted in improved product selectivity towards *p*-xylene in the catalytic alkylation of toluene.^120^ Textural properties of original and mechanically treated zeolite remained roughly similar. However, acid sites on the surface of the particles were considered largely deactivated, as cracking of bulky triisopropylbenzene could be reduced by 50%.

Improving the performance of a catalyst can be achieved by reducing the diffusion length of the reagents through the porous material. Besides the previously discussed particle size reduction, this can also be realised by introduction of larger mesopores into the microporous zeolite. Practically, this can be implemented in various ways, with recent studies showing how this can be accomplished using mechanochemical methods.

Kadja *et al.* used the same methodology as introduced by Wakihara and co-workers, where a conventionally prepared zeolite is ball milled, resulting in decreased particle size and some amorphization of the zeolite crystals, and subsequently recrystallised. In this study, the latter step is performed in the presence of CTAB (cetyltrimethylammonium bromide), thus not only the crystallinity of the material is recovered but simultaneously CTAB molecules are utilised as templates.^121^ CTAB is a common reagent used for the introduction of mesoporosity in zeolites due to its property of forming micelles in water. A calcination step after recrystallisation removes the organic template and a material with hierarchical pore structure remains. Using this methodology, the measured BET surface area was increased from 300 m^2^ g^−1^ of the parent ZSM-5 phase up to 377 m^2^ g^−1^ for a milled sample in the presence of CTAB. The micropore volume was retained, indicating that formation of mesopores was responsible for the increased available surface area, which is also supported by the shape of the adsorption isotherms.

**Fig. 7:**
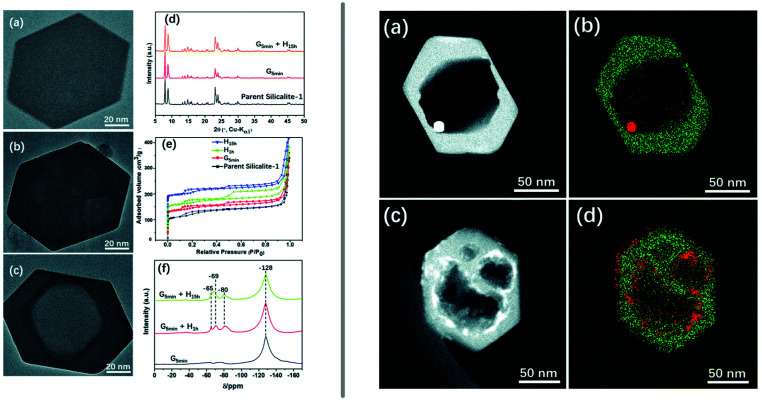
Introduction of mesoporosity into silicalite-1 (MFI) crystals by grinding the zeolite in the presence of ammonium fluoride and an organic templating agent and subsequent heat treatment. The left panel shows TEM images of a pristine zeolite crystal (a) as well as a ground example (b) and the final product (c). PXRD patterns (d), nitrogen adsorption (e), and 19F MAS NMR (f). The righthand panel depicts STEM images and elemental maps of similarly treated mesoporous silicalite-1, previously impregnated using platinum (a and b) or cobalt (c and d) precursors to produce active catalyst materials. Reproduced from ref. 122 with permission from the Royal Society of Chemistry.

Even more elegantly, Huang *et al.* showed that desired mesopores can be introduced into zeolites during the synthesis of the material itself.^122^ Hydrothermally prepared and calcined zeolite silicalite-1 (**MFI**) was manually ground with ammonium fluoride and TPA bromide (TPA = tetrapropylammonium) or CTAB, followed by heating in an autoclave at 180 °C and calcination to remove the organic compound.^122^ The resulting material exhibits additional meso-/macropores (Fig. 7 left), whose size can be tuned by adjusting the duration of the sheat treatment and by the choice of organic additive. The developed procedure was also adapted to include an impregnation step of the catalytically relevant metals platinum and cobalt prior to the milling step, which yielded nanoparticles, encapsulated in the zeolite (Fig. 7 right).

In another study, Andrade *et al.* used ball milling for loading of iron unto conventionally prepared hierarchical zeolite beta.^123^ Hydrated iron chloride was mixed with the zeolite in a planetary ball mill and the resulting material exhibited enhanced properties in the oxidation of 1-phenylethanol, conducted in a microwave. It is, however, unclear what the nature of the resulting iron species is and how well they are distributed throughout the zeolite support.

Zeolites that are prepared as purely siliceous frameworks typically require post-synthetic treatment in order to be utilisable as catalysts or ion-exchange materials. This can be done by an alumination which is for example achieved by hydrothermal treatment with an aqueous solution of an aluminium salt or atomic layer deposition using gaseous trimethylaluminium. As an alternative to these processes, De Prins *et al.* have published a low energy wet ball milling procedure in which they were able to incorporate aluminium into zeolite COK-14, a material obtained through an inverse Sigma transformation from the parent zeolite phase with framework **UTL**.^124^ Conducting the alumination with a mechanochemical method also enables a simultaneous particle size reduction and therefore shorter diffusion paths, improving the catalytic performance of the resulting material as expected.

**Fig. 8:**
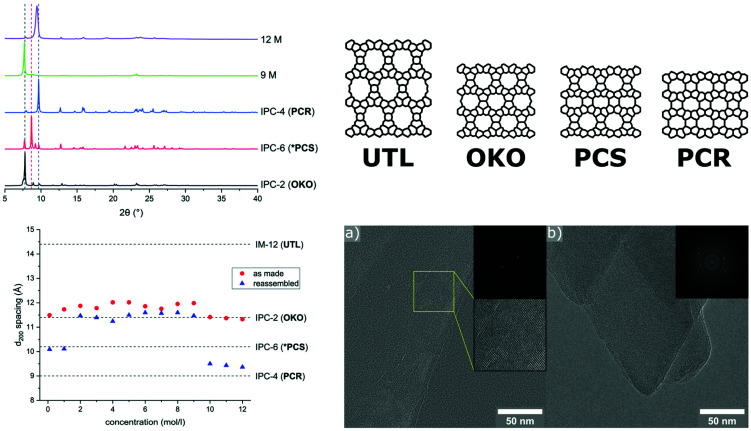
Hydrolysis of zeolite **UTL** using a ball milling assisted method using comparatively low amounts of hydrochloric acid. PXRD patterns and d_200_-spacing vs acid concentration are shown on the left-hand side. The right panel shows the known structures of the parent zeolite UTL and relevant daughter phases, as well as TEM images of materials obtained with 25 mL 12 M HCl, showing lattice fringes indicative of the PCR framework. Adapted and reproduced from ref. 126 with permission from the Royal Society of Chemistry.

A similar low energy ball milling methodology has been used in our group, assisting in the second step of the ADOR (Assembly-Disassembly-Organisation-Reassembly) process, in which a parent germanosilicate zeolite phase is hydrolysed using water or acid solutions.^125^ The ADOR process is possible because of incorporation of a ‘weakness’ into frameworks such as **UTL**, where silicon-rich layers are connected to each other by germanium-rich double 4-ring units (D4R). The germanium-oxygen bond is rather labile in water as well as acidic media, thus a controlled removal of the D4R units is possible. Therefore, the disassembly is a hydrolysis reaction, typically conducted for several hours under reflux conditions and with high liquid/solid ratios. Tuning synthesis conditions such as acid concentration, reaction time, and temperature, allows for targeting of specific final materials. These differ in their layer connectivity, ranging from direct oxygen linkages to a partially re-established single and double 4-ring units, easily recognisable by comparing the corresponding shift of the interlayer peak (d_200_) in PXRD patterns. In the mechanochemically assisted approach, known products of the ADOR process were afforded without the need for a heating source, in only 30 min and with a significantly decreased amount of liquid.^126^ Interestingly, in this new method the highest acid concentrations (12 M HCl) led to the denser **PCR** framework (Fig. 8), in contrast to the usually obtained **OKO** structure. Furthermore, it was demonstrated that reagent levels of water are sufficient for the disassembly to occur with the final product exhibiting an **OKO**-type framework. Such low quantities of water enable cost-efficient incorporation of ^17^O using H_2_^17^O for solid-state NMR investigations, which is usually unfeasible without an enrichment procedure. Especially for oxides such as zeolites, this additional method affording materials with a higher amount of the NMR active oxygen isotope should help widen the applicability of this characterisation method, as discussed recently by Ashbrook *et al.*^127^

### Perspective and outlook

Several new possibilities for mechanochemical methods have been shown to enrich zeolite science in recent years. Increasing the efficiency of syntheses, be it through the activation of the raw materials or seed crystals, thus decreasing the required time to crystallise the desired zeolite phase, improve purity or enhance its properties for a given application, is a much-desired goal. Going even one step further, in some cases it is possible to circumvent the need for a traditional solvent-based synthesis altogether and directly heat-treat the product of the milling procedure, affording highly crystalline zeolite materials. Likewise, mechanochemical methods can be used to tailor textural properties such as particle size, morphology, desired partial amorphization as well as post-synthetic introduction of mesopores, all of which lead to materials with improved properties.

Furthermore, using cheap oxide precursors catalytically active heteroatoms such as titanium or tin were successfully incorporated into zeolite frameworks through mechanochemical methods. This presents not only a welcome alternative to common preparation methods, but also hints at the possibility for the inclusion of otherwise unfeasible transition metals, be it due to the lack of applicable precursors for conventional methods or obstacles with respect to their cost or availability. Additionally, using mechanochemical methods in post-synthetic alterations has been shown to both allow alumination and stannation as well as combining production of mesoporosity with formation of transition metal nanoparticles by impregnation. The ease of accessibility to heteroatom containing zeolites is a very promising avenue and may prove crucial for the development this subclass.

Besides the classification we have used in this article of pre- and post-synthetic application of mechanical methods, one may also categorise with respect to the nature of their effects: physical or chemical. Both are relevant at all stages of an experiment, so care must be taken when assessing their influence. Physical changes involve mainly the classical particle size reduction, which is beneficial for pre-treatment of seed crystals due to an increase in total number of particles. Likewise, smaller size of raw materials may aid in terms of reactivity due to the higher surface to volume ratio. In both instances, the high degree of mixing will have a favourable effect on the synthesis. Increasing the available outer surface area of particles by decreasing their size has also a great impact when it comes to diffusion limitation, which are often encountered in catalytic reactions.

Accompanying these physical effects of mechanical forces are plenty of chemical changes which have been reported in the studies highlighted above. Almost ubiquitous is amorphization of crystals; a direct consequence of the high impact milling media has on a given sample. Although such a result is usually viewed in a negative light, one should keep in mind that the deterioration of crystallinity may only occur on the surface of the particle, whereas the bulk still exhibits long range ordering of atoms. The crystallinity of the whole particle can be recovered by chemical means using heat, while in other cases this amorphization is even advantageous for the desired application. Furthermore, mechanochemical methods have been shown to induce changes to the surface charge (zeta potential) which is of special interest in separation problems, where the control over the attraction/repulsion of molecules with respect to the surface of the zeolite-based separating membrane are of crucial importance.

Mechanical treatment can also produce oxygen radicals on zeolite crystals. This has been exploited to achieve faster synthesis of zeolites when using such radicalised seed crystals. This should be of further interest as these studies shed some light on a chemical mechanism for why and how milled seed-assisted synthesis proceeds.

Despite all the advantages listed, one aspect still eludes researchers so far: true mechanosynthesis of zeolites. Materials that share structural similarities with zeolites such as MOFs and ZIFs have been successfully prepared using mechanochemical methods. Why then, has it not been possible so far to find a way to translate the hydrothermal synthesis conditions to equivalent mechanochemical ones? In principle, it may not come as a big surprise when considering the very strict and narrow windows of synthesis conditions one must adhere to for the successful preparation of a specific zeolite. However, similar restrictions are quite often also seen in the preparation of other porous materials.

As is common for any phenomena, there is most likely not one single cause but rather an interplay between several aspects that inhibits the direct conversion of raw materials to zeolite frameworks. The type of bond created in the preparation of MOFs is a coordination bond, so decidedly different compared to the covalent silicon/aluminium–oxygen bond in zeolites. The breaking and making of these T–O bonds itself has been shown to be possible, otherwise amorphization of already prepared zeolites in a ball mill would not be possible. However, the bond formation on its own is not enough, but must also occur in a very controlled manner as there are many different T–O–T and O–T–O angles in a given zeolite framework, whereas in a typical MOF the variety of required angles of the formed bonds is much smaller. Without such control, the result is a disordered, amorphous, or dense material. On this topic, it is also important to note, that in commonly used solid silica precursors, such as fumed silica and silica gel, silicon may not exhibit the same tetrahedral coordination as in the desired zeolite framework. Several materials are reported in the literature where the synthesis works strictly with tetraethoxysilane, whereas other silicon sources such as fumed silica only led to impure products or no zeolite at all.

Usually, mechanochemical treatments generate heat due to friction, which can also lead to localised hot spots of several hundred degrees Celsius. Thus, the question arises why a high energy milling process itself does not produce a zeolite directly, while a subsequent treatment in an autoclave results in the desired material. One possible cause for this may be that despite the potential for confined areas with high temperatures, the bulk material may not be exposed to heat beyond a few degrees above room temperature. This could be remedied by combining the above described successful mechanochemical pre-treatment with heating into one single-step process, by which the desired zeolite phase can be obtained. This may be achieved by wrapping the milling vessel in a heated cover as described recently by Cindro *et al.*^128^ Similarly, it has been realised by using a heat gun aimed at the milling flask.^129^ Still, it could be argued that such a synthesis is not comparable to the conditions employed in a typical mechanochemical MOF synthesis, requiring no additional, secondary heating source. Nonetheless, such a setup may prove to be a crucial step towards the direct synthesis of zeolite products.

Another cause for unsuccessful mechanochemical formation of zeolitic products could be that crystallisation conducted in autoclaves enable autogenous pressure due to some residual water present in the mix of reagent materials. This increased pressure may be the crucial parameter enabling the formation of a porous structure over denser polymorphs. Such a condition is not met in a conventional milling apparatus, where the pressure inside the milling vessels will be approximately ambient. This may also serve as an explanation why experiments that are conducted entirely water-free (without hydrated reagents or addition of minute amounts of water) are unsuccessful as the liquid aids in generating a higher pressure within the vessel.

Another, potentially more accessible goal for the near future, is related to the delamination of layered zeolite phases by mechanochemical means. Due to their nature, such 2D zeolites have a much larger available external surface area, which may be beneficial for applications such as the catalytic conversions of large molecules.^130^ Furthermore, this subclass of zeolites may be altered post-synthetically for example by pillaring to form structurally more robust or otherwise improve materials, while retaining most of the benefits. These usages, however, hinge on the capability of separating individual lamella from each other, necessitating mostly chemical treatments. Delamination or exfoliation procedures are also a crucial part in the formation of graphene, the proto-typical 2D material. Several studies have shown that the formation of graphene sheets can be aided by mechanochemical methods, where graphite is ball milled under specific conditions and graphene in excellent quality can be obtained.^131–133^ Such a methodology may be equally applicable to zeolites, potentially simplifying the necessary procedure or improving current protocols in terms of sustainability or efficiency.

There are many reasons to be excited about the combination of mechanochemistry and zeolites when considering the continuous growth of the field over the last years. Not only are viable and efficient adaptions of conventional synthesis methods available, but also application driven improvements have been achieved and specific properties can be targeted for tailor-made products. All these advantages are also of particular interest in view of the many industrial applications of zeolites. The potential of comparatively easy upscaling of mechanochemical methods as well as their many intrinsic benefits such as low ecological impact, high efficiency and improved cost efficiency promises high applicability in both lab scale as well as mass production. We hope that the field is just getting started and, in our opinion, mechanochemical methods are deemed to be an important part of the future of zeolite science.

## Conflicts of interest

There are no conflicts to declare.
